# The significance of case detection ratios for predictions on the outcome of an epidemic - a message from mathematical modelers

**DOI:** 10.1186/s13690-020-00445-8

**Published:** 2020-07-14

**Authors:** Jan Fuhrmann, Maria Vittoria Barbarossa

**Affiliations:** 1 Jülich Supercomputing Center, Forschungszentrum Jülich, Jülich, Germany; 2 Frankfurt Institute of Advanced Studies, Frankfurt, Germany

**Keywords:** COVID-19, Detection ratio, Mathematical modeling, Simulation, Case fatality ratio

## Abstract

In attempting to predict the further course of the novel coronavirus disease (COVID-19) pandemic caused by SARS-CoV-2, mathematical models of different types are frequently employed and calibrated to reported case numbers. Among the major challenges in interpreting these data is the uncertainty about the amount of undetected infections, or conversely: the detection ratio. As a result, some models make assumptions about the percentage of detected cases among total infections while others completely neglect undetected cases. Here, we illustrate how model projections about case and fatality numbers vary significantly under varying assumptions on the detection ratio. Uncertainties in model predictions can be significantly reduced by representative testing, both for antibodies and active virus RNA, to uncover past and current infections that have gone undetected thus far.

## Background

The World Health Organization declared the outbreak of the respiratory disease COVID-19 (coronavirus disease 2019, caused by the virus SARS-CoV-2, Severe Acute Respiratory Syndrome CoronaVirus 2) a global pandemic on March 11, 2020. In the past months, numerous efforts have been undertaken to understand the properties of SARS-CoV-2 and control the spread of the disease. While it has been repeatedly observed that the disease occurs in different severities, from very mild to critical, it is yet to be clarified to what extent pre-symptomatic or asymptomatic infections do contribute to the spread of the virus. Whereas pre-symptomatic individuals are clearly contagious with the highest infectiousness reportedly being reached right before symptom onset, the jury is still out on the amount of viral particles released from asymptomatic cases and the resulting risk of transmission.

In the attempt to predict the course of the outbreak and to possibly achieve its mitigation, mathematical models have been devised to predict the course, and possible outcomes for different countries have been presented. It was noted by several authors (e.g., [1]) that an important parameter of the epidemic is the detection ratio, meaning the percentage of infections that are actually discovered. Most mathematical models are able to reproduce the chronology of case numbers under widely varying assumptions on the value of the detection ratio. In particular, as has been noted in [2], the dynamic parameters like the reproduction number $\mathcal {R}$ derived from the case number data are completely independent of the detection ratio, assuming the latter is constant over time. This assumption, however, is debatable. For example in Germany, according to reports of the Robert Koch Institute (RKI), while the number of administered tests significantly increased in mid March 2020 (from some 127,000 in week 11 to some 360,000 in week 12) and then remained at levels between 300,000 and 450,000, the number of positive cases kept declining throughout May 2020.

Since detection ratios are also notoriously hard to obtain at early stages of an epidemic, many models use overly optimistic detection ratios of 90% or more [3], or do not even take undetected cases into account at all [4, 5]. The differences in reported case numbers during the initial phase of an epidemic outbreak may not be noticeable. However, in the long run, the number of undetected infections, and in particular undetected recoveries, plays a major role in reducing the number of susceptible individuals within a population and thereby achieving the threshold of herd immunity. Though in some countries this might have been deemed a possible strategy, achieving herd immunity in Germany without a vaccine available was not seen much as an option. Moreover, as has been pointed out by, e.g., [6], the number of undiscovered infections is missing in the denominator when case fatality rates, i.e., the percentage of infected individuals dying from the disease, are being calculated. The uncertainty about detection ratios seems to be a major determinant of widely varying estimates for the mortality of COVID-19, in particular when plain case fatality rates are considered.

Unfortunately, different approaches to determining these ratios have delivered a huge range of estimates for different geographic regions, ranging from detection ratios as low as 2% [7] to approximately 35% [7, 8], and it is not obvious how much of the variation is accounted for by actual differences in detection rates between countries and how much methodology contributes to these differences. For Germany, preliminary unofficial results for Gangelt in the county of Heinsberg [9], seem to yield about 10-20% case detection. Consistently, screening for antibodies, and therefore counting recovered individuals as well as currently infected ones, seems to yield lower estimates than screening studies using PCR-tests for active virus RNA. Possible reasons may include that (i) mild infections could easily go unnoticed, or that (ii) false negative rates of PCR-tests being caused by less than perfect sample collection [10].

We have previously proposed mathematical models for the dynamics of COVID-19 infections in Germany, in particular taking into account the effect of current and possible non-pharmaceutical control measures [11]. For the simulated scenarios in our most recent work [11], we assumed detection ratios closer to the upper end of the range detailed above (close to 40%) and remarked that the predicted fatality numbers should be expected to look very different when a lower detection ratio is assumed.

To illustrate this effect, we present here the results of simulations assuming different detection ratios, while maintaining unchanged assumptions on the other basic model parameters. For the scenarios which we show below we do not take into account the limited capacity of the health care system (this factor would further aggravate the situation in scenarios with high numbers of active cases). Our simulations shall only show the time course of both *known* and *total* active cases, as well as the cumulative number of fatalities. The latter model output is not only of high importance but also particularly sensitive to assumptions on detection.

## Results and discussion

In order to illustrate how different assumptions on the detection ratio (DR) affect predictions of the epidemic’s course, we show here simulation results for a few scenarios under the assumptions of high detection ratio (*DR*≈40*%*, comparable to the one in [11]), medium detection ratio (*DR*≈10*%*), and low detection ratio (*DR*≈2.5*%*) each. These values are taken as average over the course of the epidemic, including a probable improvement in detection between calendar weeks 11 and 12 due to the significant increase in the number of tests conducted.

The model used for simulation is an extended version of the classical susceptible - exposed - infectious - recovered (SEIR) system, with three age groups and different compartments of infectious individuals (based on our previous work [11]). Case and death counts reported in Germany by the Robert Koch Institute (RKI) as of April 24, 2020 were used for model calibration. In essence, this means estimating the effective contact rates only up to the lock-down situation in force until April 19, 2020. Though lock-down measures were partially relaxed starting on April 20, 2020, this would not show in the most recent data due to both the latency time of infection and delays in reporting. Hence the parameters used for simulations after April 20, 2020 have not been obtained from the data but rather assumed for the expected effects of the relaxed restrictions. We show model simulations of the following scenarios for Germany.

A Nonmedical interventions as of April 25, 2020, including the most recent relaxation of some lock-down measures (starting April 20) and partial reopening of schools. This results in slightly increased contact rates (undoing about 20% of the original contact reductions) in the work/school and leisure realms while all else is kept as in the baseline scenario we included as **D**.
B Interventions as in **A**, plus an additional fatigue effect leading to general awareness wearing off. People are assumed to become less careful in, e.g., sanitizing hands, keeping distance in public space, or coughing/sneezing protocol. This is assumed to gradually and partially reduce the effect of general awareness by about 50% of its original effect over the course of 8 weeks.
C Interventions as in **A**, assuming increased efforts isolating known and suspected infected individuals (called *strict case isolation*, sCI, in [11]) setting in on April 27, meaning that both detected cases and putative cases (identified as recent contacts of detected cases) are quarantined, resulting in decreased contact rates. As long as the number of active cases remains relatively low, this could be a feasible strategy.
D The original baseline scenario from [11] with interventions in place as of April 14. This is a counterfactual scenario assuming the restrictions in place had not been relaxed starting on April 20 and shown for comparison purposes. Model assumptions concerning the supposed effects of these interventions on contact rates are explained in detail in [11].

**Fig. 1:**
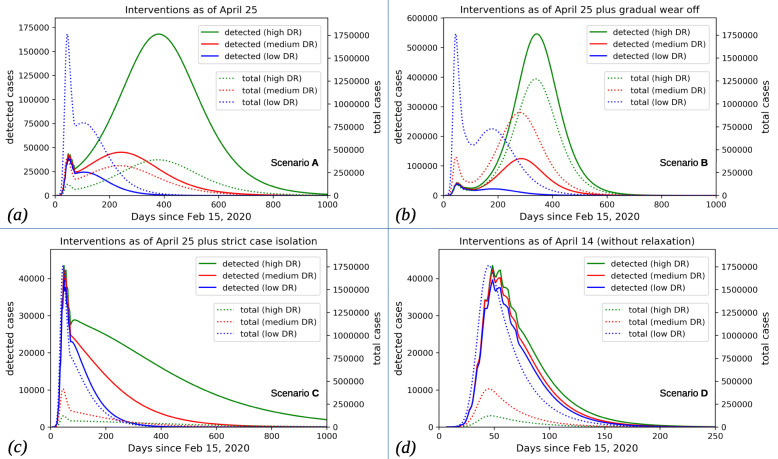
Active cases over time. Model simulations for *detected* (solid) and *total* (including asymptomatic; dotted) active cases over time for different scenarios. Notice the different scaling for detected (left axis) vs. total (right axis) cases. For each scenario, the results for high (*DR*≈40*%*), medium (*DR*≈10*%*), and low (*DR*≈2.5*%*) detection ratio are shown in green, red, and blue, respectively. Before April 20, all scenarios and detection rates yield similar results for the detected cases while total cases show the obvious differences for different detection ratios. Minor deviations in detected cases before April 20 result from slightly different fits to the weekly fluctuations in tests administered. Notice the different time scale for scenario **D** where the epidemic were to subside within a few months rather than years

Model simulations were run until the end of the epidemic, that is, until the number of active cases becomes insignificant due to the reproduction number $\mathcal {R}$ being persistently smaller than one. Note that this may be due to sufficiently low effective contact rates (cf. scenarios C and D in Fig. 1), or due to sufficiently many individuals having contracted the infection, hence having been removed from the pool of susceptibles (cf. scenarios A and B in Fig. 1). Needless to say, such long term projections are purely hypothetical since they neglect any possible reactions to the evolving situation. Specifically, it should be expected that significantly falling case numbers induce contact restrictions to be relaxed further, while rising case numbers might lead to new interventions. The precise numbers predicted by the simulations are not our main concern here. We rather want to emphasize the sensitivity of predictions to the detection ratio, that is, the different behavior exhibited by the system under the assumption of low, medium, or high detection ratios.

In Fig. 1, we show the number of active cases over time for the scenarios described above and low, medium, and high detection ratio each. For each simulation we show active *detected* cases by which we mean the number of patients having tested positive and having not yet recovered or deceased. Notice that these numbers differ significantly from the official active case numbers since the definition of recovery in official reports is based on the assumption that an individual can be safely viewed as recovered when released from hospital with no symptoms or when 14 days have passed since the positive test without the individual reporting with severe symptoms (see also "Availability of Data and Material"). The *average* duration of the infection is significantly shorter which leads to fewer active cases in the simulations since infected individuals are removed faster than officially reported. We also show the actual *total* case numbers which are all currently infectious individuals, including asymptomatic ones, independent of detection. The effect of different detection ratios is most striking in scenarios **A** (Fig. 1a) and **B** (Fig. 1b). At the time of writing this text, after having relaxed some of the measures, the reproduction number, $\mathcal {R}$, in Germany appears to be close to one, and the system is very sensitive to the proportion of susceptibles among the population. A high detection ratio implies that only a very limited number of infections remained undetected, and given the current number of detected cases, most individuals are still susceptible. In contrast, a low detection ratio suggests that a significant number of infections has been going on unobserved, and there would already be a significant number of recovered, hence immune, individuals. This can make the difference between $\mathcal {R}>1$, leading to a second peak, or $\mathcal {R}<1$ and the epidemic subsiding. In both other scenarios (**C** and **D**, shown in Fig. 1c and Fig. 1d, respectively) the reproduction number is overall smaller than in **A** and **B** for all assumed detection ratios. We therefore primarily observe a quantitative difference. Again, in these scenarios, assuming a low detection ratio means that more susceptibles could have already turned into recovered individuals, making $\mathcal {R}$ even smaller, and therefore leading to the epidemic subsiding faster. The smallest effect of variations in the detection ratio is observed in scenario **D**. In this scenario very low effective contact rates were maintained over time even after April 20, 2020. This fact reduces $\mathcal {R}$ to such small values that the lower number of susceptibles in the low *DR*-case does not make a significant difference.

**Fig. 2:**
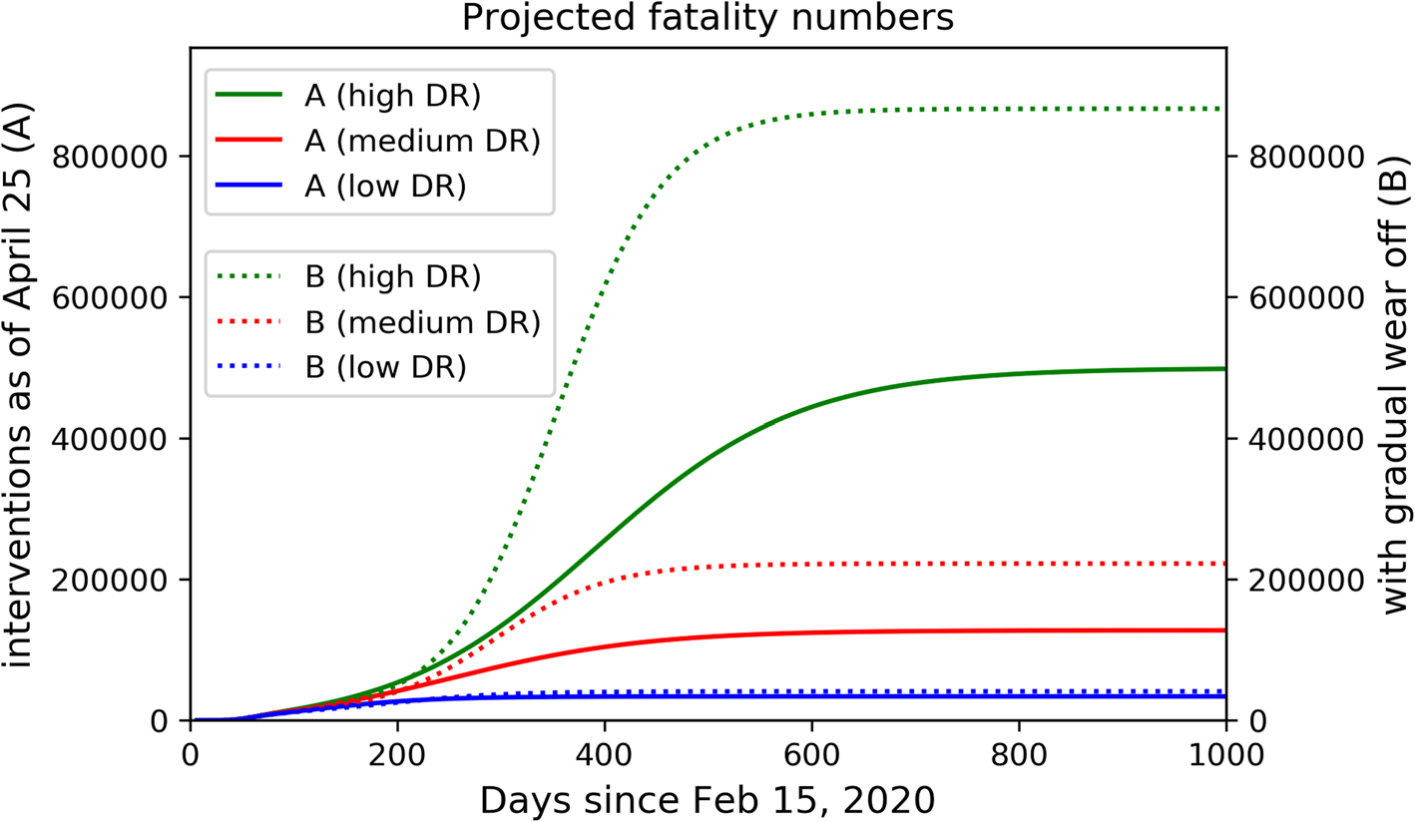
Projected fatality numbers. Projected cumulative fatality numbers under scenarios **A** (solid) and **B** (dotted) for different assumptions on the detection ratio. It is obvious that assuming a lower detection ratio leads to lower predicted total fatality numbers

Noteworthy is the effect of different detection ratios on the projected fatality number over the course of the epidemic. These numbers are obtained from the simulations by assuming a constant infection fatality rate for each age class, meaning that the same percentage of infected individuals die, independent of the actual detection rate. Parameters were set to fit reported death counts until end of March 2020 (taking into account the significant reporting delay for fatalities). The fatality rate among undetected (symptomatic) individuals is assumed to be significantly smaller than among detected ones, as it is feasible that critical cases are more reliably detected. In scenarios **A** and **B**, the assumption of high or medium detection ratios lets us predict a second peak of the epidemic, over the course of which many more fatalities are to be expected. These would not be predicted if a low detection ratio were assumed (Fig. 2). The situation is aggravated by the fact that a low detection ratio means that the case fatality rate (percentage of fatalities among detected cases) is considerably higher than the actual lethality (or infection fatality rate, cf. [6]) of the disease. Projecting observed case fatality rates into the future therefore produces overestimations of total fatality numbers. The overestimation becomes more pronounced the lower the detection ratio.

## Conclusion

While enormous efforts are undertaken all over the globe to stop the COVID-19 pandemic and limit its consequences, more and more studies indicate that a large portion of cases could have remained undocumented [12]. Here we showed how the knowledge of the detection ratio of COVID-19 infections is of crucial importance for model-based predictions on the further course of the outbreak and its control. This emphasizes the urgent need for screening representative samples of the population in order to determine the prevalence of antibodies against SARS-CoV-2. Studies like those reported in [9] for Germany or in [13] for California are promising first steps but more widespread screening with selection processes minimizing biases are necessary to obtain better estimates of past detection ratios. Continuous indiscriminate testing of individuals for virus RNA may further help to uncover temporal changes of detection ratios. It remains to be determined whether wide range screening would also help limiting the spread of the disease [14]. We conclude by noting that the difficulties in predicting an outbreak outcome are not limited to COVID-19 but pertain to any novel infectious diseases, making it even more important to not forget this lesson even after the COVID-19 pandemic will have been resolved.

## Data Availability

The publicly available dataset provided by the Robert Koch Institute (RKI https://npgeo-corona-npgeo-de.hub.arcgis.com/datasets/dd4580c810204019a7b8eb3e0b329dd6_0/data) was used for this study. The underlying model used to produce simulations shown in this study is detailed in our previous work [11]. At https://vfs.fias.science/d/7f8fd49abf, we have provided the Python code and more images for a dramatically reduced model (without age classes or stages of infection) describing a hypothetical disease. Despite the significantly lower complexity, the main effects explained in “Results and discussion” section are present as well, indicating their robustness. The model equations and a detailed presentation of the assumptions are also given there.

## Abbreviations

COVID-19: Coronavirus induced disease 2019
DR: detection ratio
PCR: polymerase chain reaction
RNA: ribonucleic acid
SARS-CoV-2: Severe acute respiratory syndrome Coronavirus 2
SEIR: susceptible - exposed - infectious - recovered (model)
